# Effects of 4-Week Intervention with* Ulmus macrocarpa* Hance Extract on Immune Function Biomarkers in Healthy Adults: A Randomized Controlled Trial

**DOI:** 10.1155/2018/5690816

**Published:** 2018-02-25

**Authors:** A Ra Cho, Sang Yeoup Lee, Young Hye Cho, Cheol Min Kim, Sung Goo Kim

**Affiliations:** ^1^Department of Family Medicine, Obesity, Nutrition and Metabolism Clinic and Research Institute of Convergence of Biomedical Science and Technology, Pusan National University Yangsan Hospital, Yangsan 50612, Republic of Korea; ^2^Department of Medical Education, Pusan National University School of Medicine, Yangsan 50612, Republic of Korea; ^3^Department of Biomedical Informatics, Pusan National University School of Medicine, Yangsan 50612, Republic of Korea; ^4^Center for Anti-Aging Industry, Pusan National University, Busan 47046, Republic of Korea; ^5^Bio-Port Korea INC, Marine Bio-Industry Development Center, Busan 46048, Republic of Korea

## Abstract

*Ulmus macrocarpa* extract has been shown to have immune-related effects in animals, but no studies have yet been performed in humans. This randomized, double-blind, placebo-controlled trial was conducted to determine the effect of short-term administration of* Ulmus macrocarpa* Hance extract (UME) on immune function biomarkers and its safety in human subjects. Fifty-eight subjects were randomly assigned to a UME group or a placebo group. Subjects in the UME group were given 500 mg per day of UME orally for 4 weeks. Mean fluorescence intensity (MFI) of tumor necrotic factor-*α* increased only in the UME group at 1 week (*P* = 0.027). The MFI of interleukin-2 decreased less significantly in the UME group than in the placebo group at 1 week (*P* = 0.028). However, unfortunately, at 4 weeks, no intergroup differences were detected in MFIs of cytokine. In conclusion, administration of UME for 1 week increased serum TNF-*α* and sustains IL-2 in human, which suggests that UME increases Th1-related immune function in the short term in healthy people. However, additional studies are needed to confirm the results of this first-stage study and further trials are required to decide on optimal dosage and duration of administration. This trial is registered with ClinicalTrials.gov Identifier: NCT02414412.

## 1. Introduction

Tremendous advances in medical technology have nearly doubled life expectancy over the past century [1]. Previous studies have reported positive relationship between immune status and longevity and suggested that an impaired immune system adversely affects life expectancy [2]. In addition, abnormal circadian rhythms, industry, modern life, a sedentary lifestyle, and unhealthy diets have been linked with depression of the immune system, obesity, and diabetes mellitus. For this reason, considerable efforts are being made to enhance the immune system [3]. Immune response can be classified as innate or adaptive. These responses involve complements, various cytokines and macrophages, natural killer (NK) cells, and lymphocytes, and reduced activities of these cells are associated with reduced immune system effectiveness [4].

*Ulmus macrocarpa* Hance, otherwise known as the large-fruited elm, is a deciduous tree or large shrub endemic to Korea, Japan, and China. The bark of its stems and roots are traditionally used to treat of swelling, mastitis, inflammation, and stomach disease [5]. Previous animal studies have reported that* Ulmus macrocarpa* Hance extract (UME) has several immune-related effects, for example, nitric oxide-producing properties, antibacterial effects, and enhancement of immune function in rats [6–8]. However, no study has been performed on the effects of UME on immune function in human. Therefore, we conducted this randomized, double-blinded, placebo-controlled trial to determine the effect of the short-term administration of UME on immune function biomarkers in human.

## 2. Materials and Methods

### 2.1. Study Design and Subjects

This randomized, double-blind, placebo-controlled, parallel-group trial was approved by the Institutional Review Board of Pusan National University, Yangsan Hospital (IRB number 02-2014-031), and was performed in accordance with the principles of the Declaration of Helsinki. Written informed consent was obtained from all study subjects, which were recruited by advertising at a tertiary hospital. This trial was registered at clinicaltrials.gov as NCT02414412.

### 2.2. Eligibility Criteria

The eligibility criteria applied were age of 19–65 years and a peripheral blood WBC of 4,000–8,000/mm^3^, suggesting a person with a healthy immune system. On the other hand, subjects with a history of fracture during the previous year; with an abnormal liver or renal function (serum aminotransferase ≥ 60 IU/L, serum creatinine ≥ 1.2 mg/dL, or proteinuria, defined as a urinalysis dipstick reading of ≥2+); with diabetes mellitus (diagnosed clinically or a fasting glucose of >126 mg/dL); with uncontrolled hypertension or a notable cardiac disease history, such as angina or myocardial infarction; with a history of gastrectomy; who are diagnosed with immune disease; or who are taking herbs or medication for psychiatric disease or any medication that might influence immune function within the previous 4 weeks were excluded.

### 2.3. Interventions

Subjects were randomly assigned to the UME (supplied by Bio-Port Korea Inc., South Korea) or the placebo group. Subjects randomized to the UME group were given 500 mg per day of UME orally, that is, one 250 mg tablet 30 minutes after breakfast and another one 30 minutes after dinner for 4 weeks. Members of the placebo group were given the same quantity of placebo in an identical manner.

### 2.4. Ingredients of UME

The mean level of total catechin contents in the UME was 5.08 mg/g (range, 4.06–6.09 mg/g). The proportions of each catechin are (−)-epigallocatechin (37.19%), (−)-epigallocatechin-gallate (3.58%), (−)-epicatechin (38.04%), and (−)-epicatechin-gallate (21.19%).

### 2.5. Outcomes

The primary study outcome measure was a change in NK cell activity over the 4-week treatment period. Secondary outcome measures were changes in peripheral white blood cell (WBC) count and tumor necrosis factor-*α* (TNF-*α*), interferon-*γ* (IFN-*γ*), interleukin- (IL-) 1*β*, IL-2, IL-4, IL-8, IL-10, and IL-12.

### 2.6. NK Cell Activity

Cytotoxic activity of NK cells was measured using the NK Vue-Kit® (ATgen, Seongnam, Republic of Korea). Whole blood (1 ml) was collected in NK Vue® Tube and incubated for 24 hours at 37°C. Cell-free supernatants were harvested, and IFN-*γ* values were determined according to manufacturer's protocols.

### 2.7. Cytokine Assays

Serum cytokines (TNF-*α*, IFN-*γ*, IL-1*β*, IL-2, IL-4, IL-8, IL-10, and IL-12) were measured using the human magnetic Luminex® screening assay kit (R&D Systems, Minneapolis, MN) on the bio-plex200 platform (Bio-Rad, Hercules, CA, USA). Blood samples (8 mL) collected from each patient were maintained in serum separator tubes and immediately stored at −80°C in polypropylene tubes. On the day of assay, frozen plasma was thawed, mixed by vortexing, and then centrifuged at 10,000 rpm for 5 min to isolate debris prior to use in the assay. All assays and procedures were conducted according to the manufacturer's instructions using a handheld magnetic separator block for 96-well flat bottom plates (Millipore, Millipore Corp., Billerica, MA) and analyzed using the Luminex 200 system (Bio-Rad Corp., Hercules, CA).

### 2.8. Randomization

Simple randomization to the two study groups was performed using a random numbers table. Participants were assigned randomization numbers sequentially at study recruitment; the randomization codes were held by the company who had manufactured the UME and the dummy placebo. The person that decided whether participants would be included in the study and those that performed measurements were unaware of randomization assignments.

### 2.9. Study Visits

Subjects were seen at a clinic by a physician at baseline (0 week), 1 week (±3 days), and 4 weeks (±5 days) from study commencement. At each visit, the patient history was taken and a physical examination and laboratory tests were performed. At the 1- and 4-week visits, subjects presented daily symptom diaries detailing side effects and upper respiratory tract infections, such as cough, running or congested nose, sore throat, stiffness or chills, fever, achiness, or headache. Potential adverse effects were monitored by measuring aspartate transaminase (AST), alanine transaminase (ALT), and creatine kinase (CK) and by conducting electrocardiography. Blood samples were obtained at 08:00 a.m. from an antecubital vein after a 12 h fast at baseline (0 week), 1 week (±3 days), and 4 weeks (±5 days).

### 2.10. Adverse Parameters

Serum creatinine was measured using Jaffe's kinetic alkaline picrate method and pyruvates were measured enzymatically. Serum CKs were determined by the UV kinetic method. Serum ASTs and ALTs were determined using an enzymatic colorimetric method (Toshiba TBA200FR Biochemical Analyzer, Toshiba Co. Ltd., Tokyo, Japan).

### 2.11. Statistical Analysis

Study sample size was calculated based on the ability to detect a 40% difference in peripheral blood NK cell activity after intervention. Assuming a deviation of 12% from the results obtained in a previous study [3], we estimated that 23 subjects per group would be required (two-tailed *α* = 0.05, *β* = 0.20). To account for a potential dropout rate of 20%, a total of 73 subjects were screened, but finally 58 subjects were enrolled and randomized. Efficacy analysis was performed on an intent-to-treat (ITT) basis on subjects that received at least one dose of UME or placebo. If the outcome value was missing for the participant, we inserted the last recorded value for that outcome (i.e., last observation carried forward). Normally distributed data (as determined using the Shapiro-Wilk test) were expressed as means and standard deviations. Intragroup comparisons were performed using the paired *t*-test. A repeated measure ANOVA was used to analyze intragroup changes. Intergroup comparisons were performed using the two-sample *t*-test or the Mann–Whitney *U* test for continuous variables or the chi-square test/Fisher's exact test for categorical variables. Statistical significance was accepted for *P* values < 0.05, and SAS ver. 9.3 was used for the analysis.

## 3. Results

**Figure 1:**
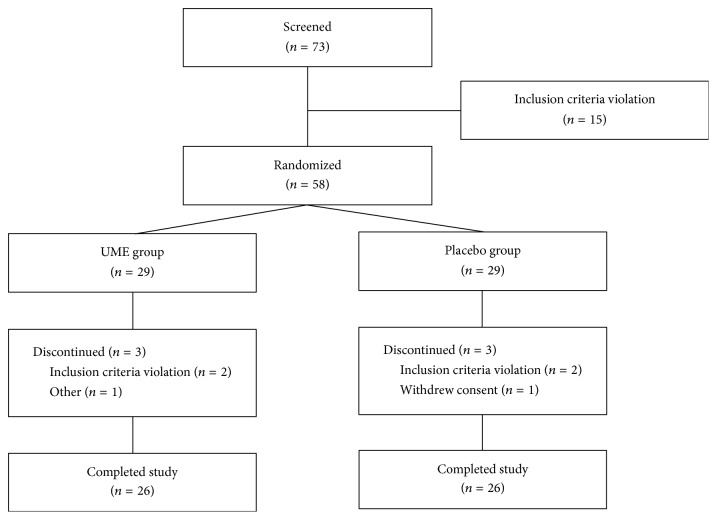
Participants flowchart (*N* = 58) showing the entire 4-week study period. UME,* Ulmus macrocarpa* Hance extract.

58 subjects were randomly assigned into two groups, UME group (*n* = 29) and placebo group (*n* = 29). Three subjects discontinued use of UME (2 subjects, other medicine; 1 subject, low compliance) and 3 subjects discontinued use of placebo (2 subjects, other medicine; 1 subject, withdrawing consent). 26 subjects in the UME group and 26 subjects in the placebo group attended 1- and 4-week visits (Figure 1). The average compliance rate of the 58 subjects was 89.7%. The UME and placebo groups were comparable with respect to most variables; no significant intergroup differences were observed between baseline demographics, anthropometrics, or body temperatures (Table 1).

Both groups exhibited no change in NK cell activities or peripheral blood WBC counts during initial 1 week (Table 2). At 1 week, mean fluorescence intensity (MFI) of IFN-*γ* was significantly lower than baseline in the placebo group (*P* = 0.007), and the MFIs of IL-2, IL-4, and IL12 also were lower than baseline in the placebo group (*P* = 0.001, *P* = 0.010, and *P* = 0.039, resp.). In contrast, the MFI of IL 8 was higher in the placebo group than at baseline (*P* = 0.023, data not shown).

Table 2 shows the differences in the change of NK cell activities and WBC counts between the groups during the experimental period. Repeated measures ANOVA showed no significant main effects of intervention and time. UME had no significant effect on NK cell activity or peripheral WBC. Repeated measures ANOVAs (1-week *x* group) demonstrated a significant difference between groups in pre-to-post intervention changes in MFI of TNF-*α*  (*P* = 0.026) and IL-2 (*P* = 0.035, data not shown).

NK cell activities increased at 4 weeks after study commencement in both groups. The MFIs of TNF-*α* and IL-10 were lower in the placebo group than at baseline (*P* = 0.044 and *P* = 0.018, resp.). The MFIs of IFN-*γ*, IL-1*β*, IL-2, IL-4, and IL-12 were lower in both groups than at baseline. In contrast, the MFI of IL 8 was higher in the placebo group than at baseline (*P* = 0.006). However, there was no difference on NK cell activities between the groups. Also, no significant effect of UME supplementation over time in MFI of TNF-*α* and IL was detected between the groups at week 4 (data not shown).

UME was well tolerated and no notable adverse effects were reported. The mild adverse effects reported were sleepiness, diarrhea, abdominal pain, herpes labialis, and an upper respiratory symptom (one subject each) in the UME group, and dry mouth (*n* = 1), diarrhea with abdominal pain (*n* = 2), constipation (*n* = 1), and epigastric pain (*n* = 1) in the placebo group. No changes in liver function, renal function, or CK were observed in either group (data not shown).

## 4. Discussion

The purpose of this study was to determine the effects of oral UME for short periods on immune function biomarkers in human subjects. The study shows that the MFI of TNF-*α* increased after 1 week of UME administration, but not after placebo. In addition, at 1 week, the MFI of IL-2 reduced less in the UME group than in the placebo group. However, unfortunately, at 4 weeks, no intergroup differences were detected in MFIs of cytokine.

Although several studies have examined the effects of dietary supplements on immune function in man [9, 10], few have evaluated relationships between medicinal plants and immune function in human subjects. A recent systematic review and meta-analysis demonstrated the beneficial effects of plant flavonoids on upper respiratory tract infections and immune function [11]. Another clinical trial reported that the water extract of the roots of* Eurycoma Longifolia* favorably influenced immune parameters, such as total T cell, CD4^+^ T cell, and naïve T cell counts [12].

*Ulmus* genus is deciduous tree of the Ulmaceae family, which is widely grown in Asia. In particular,* Ulmus davidiana* var.* japonica* (elm bark) extracts are known to increase immunocompetence, that is, to enhance splenocyte proliferation and cytokine production capacity by activated macrophages in mice [13]. In one study, it was shown that* Ulmus davidiana* var.* japonica Nakai* extracts have antioxidative activities, antiapoptotic effects, and inhibitory effects on DNA synthesis and cytokine production in mouse immune cell cultures [14]. In another study on* Ulmus macrocarpa* Hance, it was suggested that UME had an immune-modulatory effect in a mouse model, because serum IL-2, IL-12, and IFN-*γ* were elevated in a UME-fed group [8]. However, previous studies [8, 13, 14] have been conducted in animals, whereas the present study is the first work to investigate the effect of UME on immune function in human subjects.

**Figure 2:**
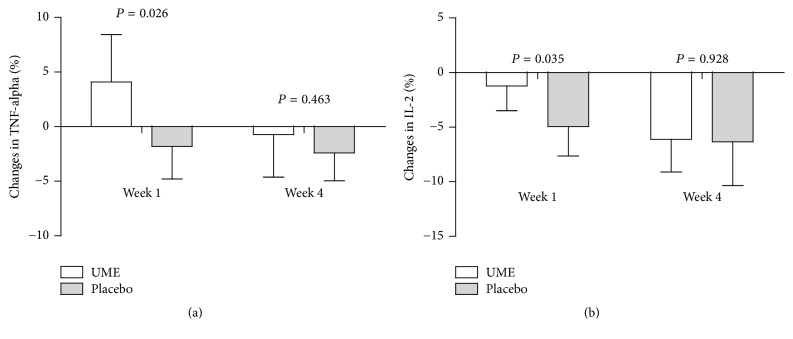
Changes (%) in tumor necrosis factor-*α* (a) and interleukin-2 (b) levels of the two study groups. Data are expressed as means with 95% CI. Percentage change was calculated by subtracting the baseline value from each posttreatment value, dividing the difference by the baseline value, and multiplying by 100. *P* values were calculated based on the two-sample *t*-test.

Cytokine production is attributed to two broad categories dependent on the functional profiles of the secreting T-helper cells. Type 1 helper cells (Th1) generally mediate cellular immune response through the activities of cytotoxic lymphocytes, NK cells, and macrophages and produce interferon, TNF-*α*, and IL 2. On the other hand, type 2 helper cells (Th2) enhance immune reactions mediated by antibodies and produce IL 4, IL 5, IL 6, and IL 10 [15]. Unfortunately, we did not observe the differences between the UME and placebo groups in changes of NK cell activities or peripheral WBC counts during the 4-week experimental period. However, the MFI of TNF-*α* (4.1%) increased only in the UME group at 1 week (Figure 2(a), *P* = 0.026). The MFI of IL-2 decreased less significantly in the UME group (−1.8%) than in the placebo group (−2.4%) at 1 week (Figure 2(b), *P* = 0.035). These finding suggest that UME might enhance Th1-related immune response in human for at least one week [15, 16]. It is the first study to evaluate the effect of UME on human immunity. Therefore, we do not know why Th1 cytokines increased at 1 week and disappeared at 4 weeks. However, as mentioned earlier, there are too many variables to control the long-term results, so no significant difference between the two groups at 4 weeks can be concluded that there is no long-term effect of UME.

Our study has several limitations, including a relatively small UME dosage. In the majority of rat and mouse studies,* Ulmus* extracts were administered at 300–600 mg/kg daily, whereas in the current human study, we administered 500 mg per day, which is equivalent to only 100 mg/kg/day of UME in rats and mice. This small dosage was used to minimize the possibilities of adverse effects and toxicity [17, 18]. In this study, NK cell activity was measured using the NK Vue-Kit (ATgen, Seongnam, Republic of Korea), which provides an easy new way to activate NK cells but still has a problem that NK cell activity has not been easily reproducible. So, it is considered that there is no difference between the UME and placebo groups, rather than the increase of the two groups in the first week in this study. Immune function is a complex process involving multiple factors, and in the present study, we can thoroughly control over initial period (1 week) but could not perform thoroughgoing control for comorbid conditions, such as infections and inflammations during the extended term of administration (4 weeks), although we measured body temperature which might reflect infection or inflammation between the two groups. We are therefore able to focus on long-term effect of UME and nevertheless, to the best of our knowledge, this is the first study to examine effect of UME on immune function in human.

In conclusion, administration of UME for 1 week increased serum TNF-*α* and sustains IL-2 in human, which suggests that UME increases Th1-related immune function in the short-term in healthy people. However, long-term effect of UME was not proven. Therefore, we suggest additional studies be undertaken to confirm the results of this first-stage study and that trials be conducted to determine optimal dosages and durations of UME administration with respect to enhancing immune function in human.

## Figures and Tables

**Table 1:** Baseline characteristics of the two study groups^1^.

	UME (*n* = 29)	Placebo (*n* = 29)	*P* value^2^
Age (years)	40.3 ± 9.4	36.2 ± 9.1	0.093
Males (%)	3 (10.3)	6 (20.7)	0.470^*∗∗*^
SBP (mmHg)	115.7 ± 13.9	113.3 ± 8.8	0.447
DBP (mmHg)	75.3 ± 9.6	73.7 ± 8.8	0.541
Waist circumference	74.9 ± 9.5	73.7 ± 8.4	0.609
Smoker (%)	0	1 (3.5)	0.670^*∗∗*^
Alcohol consumption (%)	11 (37.9)	10 (34.5)	0.785^*∗*^
Body temperature (°C)	36.4 ± 0.4	36.5 ± 0.4	0.221

^1^Values are expressed as means ± SDs or as frequencies (percentages). ^2^*P* values were determined using the ^*∗*^two-sample *t*-test or chi-square test or ^*∗∗*^Fisher's exact test. DBP, diastolic blood pressure; SBP, systolic blood pressure; and UME, *Ulmus macrocarpa *Hance extract.

**Table 2:** NK cell activities and WBC counts over a 1-week study^1^.

Variables	Group	Baseline	1 week	Difference	*P* value^*∗*^
		Mean ± SD	Mean ± SD	Mean ± SD	
NK cell activity (pg/mL)	UME (*n* = 29)	783.1 ± 378.3	921.5 ± 535.5	138.4 ± 395.8	0.072
	Placebo (*n* = 29)	868.4 ± 514.1	977.4 ± 627.1	109.0 ± 309.6	0.068
	*P* value^*∗∗*^			0.754	

White blood cell (10^−3^/*μ*L)	UME (*n* = 29)	5.3 ± 1.2	5.4 ± 1.0	0.0 ± 1.1	0.892
	Placebo (*n* = 29)	5.9 ± 1.3	5.6 ± 1.2	−0.3 ± 1.2	0.276
	*P* value^*∗∗*^			0.370	

^1^Values are expressed as means ± SDs. *P* values were determined using the ^*∗*^paired *t*-test for comparison of difference from baseline and ^*∗∗*^a repeated measure ANOVA for comparison of within-group changes. NK, natural killer; UME, *Ulmus macrocarpa *Hance extract.
